# Physical and Mechanical Characteristics of Gelatin-Based Films as a Potential Food Packaging Material: A Review

**DOI:** 10.3390/membranes12050442

**Published:** 2022-04-19

**Authors:** Nurul Saadah Said, Norizah Mhd Sarbon

**Affiliations:** Faculty of Fisheries and Food Science, Universiti Malaysia Terengganu, Kuala Nerus 21030, Malaysia; nurulsaadah.said@gmail.com

**Keywords:** food packaging, biodegradable polymer, gelatin, composite film, characterization

## Abstract

This review discusses the potential application of gelatin-based film as biodegradable food packaging material from various types of gelatin sources. The exploitation of gelatin as one of the biopolymer packaging in the food industry has rising interest among researchers as the world becomes more concerned about environmental problems caused by petroleum-based packaging and increasing consumer demands on food safety. Single gelatin-based film properties have been characterized in comparison with active and intelligent gelatin-based composite films. The physical properties of gelatin-based film such as thickness, color, and biodegradability were much influenced by total solid contents in each film. While, for mechanical and light barrier properties, poultry-based gelatin films have shown better properties compared to mammalian and marine gelatin films. This paper detailed the information on gelatin-based film characterization in comparison with active and intelligent gelatin-based composite films. The physical properties of gelatin-based film such as color, UV-Vis absorption spectra, water vapor permeability, thermal, and moisture properties are discussed along with their mechanical properties, including tensile strength and elongation at break.

## 1. Introduction

The increasing concern and interest among consumers regarding health, nutritional value, food safety, and environmental problems have spurred the development of biodegradable films. Issues on environmental pollution and exhaustion of natural resources have risen as these synthetic packaging materials possess non-biodegradability characteristics. Non-renewable resources due to the increase in energy demand are causing climate change and depletion of fossil resources since their regeneration involves the passage of many years. Despite their excellent properties, high mechanical strength, low cost of manufacturing scale, and process optimization, these materials cause significant environmental impacts in terms of greenhouse gas emissions and land and water footprints [1]. Given these considerations, an alternative way that utilizes the source of biopolymer has come into the spotlight herein solving environmental burdens and benefits of various end-of-life treatments for plastic film waste. 

Biodegradable films have been broadly studied for their potential in protecting food materials and act as a barrier towards moisture, gas, aromas and solute transfers, while offering advantages such as non-toxic materials and low-cost production [2,3,4]. At present, current trends in biopolymer as food packaging has diversified the types of material which include natural agents, plant extracts, and nanomaterials. Active, intelligent, along with nano technologies can work synergistically creating multipurpose food-packaging system while inferring good compatibilizing effect and performed their task as good packaging material [5].

Biodegradable films are commonly made from renewable sources such as protein, carbohydrates, and lipids. Proteins have been extensively studied, as they exhibit valuable traits in biodegradable film production owing to their abundance, good film-forming ability, transparency, and excellent barrier properties against O_2_, CO_2_, and lipids [2,3,4]. Thus, many researchers have been keen to study protein-based films from various protein sources such as collagen [6], gelatin [7,8], wheat gluten [9], and whey [10].

Gelatin-based packaging films have already been proposed to protect, maintain, or extend the shelf-life of food products, as they exhibit good film-forming ability and are able to act as outer films in securing food from exposure to light and oxygen [11,12,13,14]. However, due to the emergence of advanced technologies, along with changes in consumer preference for safe food, gelatin-based film has been proposed to extend its application as active and intelligent biodegradable packaging [4]. Numerous studies have been done on active gelatin films such as gelatin/carvacrol composite film, which has been found to exhibit excellent antibacterial activity against *S. aureus*, *B. subtilis*, *E. coli*, and *P. aeruginosa* [15]. Gelatin/*Centella asiatica* extract composite film has been revealed to have good antioxidant activity [16,17]. Meanwhile, among intelligent gelatin-based film, findings by Musso, Salgado, and Mauri [18] have shown that gelatin films with incorporation of three acid-base synthetics indicators, namely methyl orange (MO), neutral red (NR), and bromocresol green (BCG), have resulted in different color changes of film after contact with media of different pH levels. Active and intelligent gelatin-based films are often characterized based on physical (color, UV-Vis absorption spectra, water vapor permeability, thermal properties, and moisture properties) and mechanical (tensile strength and elongation at break) properties. This paper aims to provide detailed information on gelatin-based film characterization in terms of each film’s properties while creating awareness among consumers and retailers of the benefits of gelatin film production related to food safety and as an alternative for petroleum-based packaging.

The concept of this research originates from the major degradation problem in food packaging that were caused by utilization of synthetic polymer. Thus, this study focuses on discussing natural biodegradable multilayer film that are derived from gelatin from various sources (mammalian, marine, and poultry) with incorporation of additional substances such as natural extracts, essential oils, and nanoparticles. The section is divided based on a comparison between the single gelatin film with composite gelatin film and see how the compatibilism reactions of these composite film’s combination affect the mechanical and functional properties of the multilayer film. Thereby, it is anticipated for this combination to migrate to the interface and perform the tasks differently inside the packaging. Furthermore, this research extensively studied and highlighted which gelatin films have the best quality in each different film’s properties. On the other hand, current review trends related to gelatin films tend to focus on gelatin application in food industry and specific multilayer gelatin films discussion with certain additional substances incorporated into the films [19,20,21].

**Figure 1:**
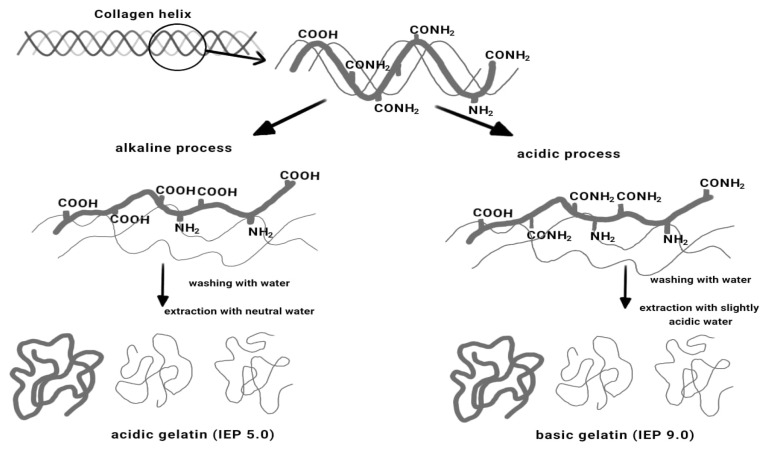
Two methods for gelatin extraction from tissues containing collagen [26].

Gelatin is a protein obtained by hydrolyzing the collagen found in the bones and skin of animals. The physical and chemical properties of gelatin are greatly affected by the source animal, age of the animal, collagen type, and extraction method used [22]. Global gelatin production was 348.9 kt in 2011 and was expected to reach 450.7 kilo tons in 2018, growing at a compound annual growth rate (CAGR) of 3.73% from 2012 to 2018 [23]. Gelatin can be classified into two types as determined by gelatin pretreatment during the extraction process. Type A gelatin with an isoionic point of 6–9 is obtained from an acid-treated precursor, whereas type B gelatin with an isoionic point of 5 is derived from an alkali-treated precursor (Figure 1). Type A gelatin was reported to have more amino acid content with higher amount of hydroxyproline, threonine, cystein, lysine, glycine, proline, alanine, leucine, and isoleucine compared type B gelatin [24].

The quality of gelatin is determined by gel strength and viscosity. Gel strength, also known as “bloom” value, is an indicator of the strength and stiffness of the gelatin. It reflects the average molecular weight of gelatin constituents, and is commonly between 30 and 300 bloom (<150 for low, 150–220 for medium and 220–300 for high bloom). A higher bloom value indicates greater gelatin strength. A different bloom value for gelatin is applied based on the type of product required and its functions [25]. 

Additionally, gel strength, gelatin concentration, pH, and temperature directly affect the viscosity of gelatin [27]. Gelatin is a nearly tasteless, odorless substance commonly produced in a granulated or powdered form. Although it has hygroscopic properties, its water absorbing capacity depends on the relative humidity at which it is dried and stored. Extreme pH and high temperature can denature gelatin and change its properties by disrupting the three-dimensional structures of gelatin and forming a random coil, resulting in lower viscosity and elastic modulus of gelatin. Therefore, the processing conditions of gelatin need to be carefully controlled to get a high gelling strength [28]. Gelatin has been globally utilized in photographic, cosmetic, and pharmaceutical industries due to its gel-forming properties. In addition, gelatin also has numerous uses in the food industry such as emulsifiers, foaming agents, colloid stabilizers, biodegradable film-forming materials, and microencapsulating agents [22]. Gelatins can be derived from numerous types of collagen sources such as porcine [29], bovine [30], fish [31], poultry [14], and insect [32] sources, among others.

## 2. Sources of Gelatin

### 2.1. Gelatin from Mammalian Sources

The most abundant gelatin sources are mainly derived from mammals, especially cattle and pig, at 46% for pig skin, 29.4% for bovine hide and 23.1% for pork and cattle bones [22]. Bovine and porcine skin gelatins are widespread across food manufacturing industry due to their high availability. Generally, gelatin from bovine skin is known as type B gelatin and is produced from alkaline treatment, while porcine skin gelatin is known as type A gelatin and is produced from acidic treatment [30] with isoelectric point of pH 4.8–5.5 and pH 7–9.4, respectively [33]. Bloom values from pig skin and bovine skin gelatin have been reported in the range of 130–308 g [34,35] and 227–350 g [36,37], respectively. The viscosity values from pigskin gelatin range from 6.37 to 7.28 cP [35] and viscosity value of bovine skin gelatin has been reported at 3.90 cP [37]. The amino acid composition of porcine gelatin was found higher in glycine, proline and arginine contents as compared to bovine gelatin [30]. Mammalian gelatin is more popular than other sources due to their superior gel qualities (gel strength and viscosity) and strong film forming properties. Additionally, numerous studies have been conducted on active and intelligent mammalian gelatin, films such as active bovine gelatin/nano chitin/corn oil composite film [38] and intelligent bovine/curcumin composite film [39].

However, mammalian gelatins have major drawbacks and issues regarding religious concerns and Halal issues, as they cannot be used or consumed by Muslims, Jews, or Hindus on various grounds [40]. Moreover, due to the potential risk in spreading harmful pathogens derived from bovine spongiform encephalopathy (BSE), also known as mad cow diseases and food and mouth disease (FMD), alternative gelatin sources for porcine and bovine gelatin substitution have been given priority and taken into consideration [3]. The utilization of alternatives gelatin from various sources has become greatly advantageous for the food industry due to rapidly growing interest in the global market for halal certified food [40]. New gelatin sources from marine species such as shortfin scad skin [41], giant squid skin [42] and eel skin [31,43]; poultry sources like chicken skin, feet, and bone [14,44] and duck feet [45] have increased researcher interest in replacing mammalian resources.

### 2.2. Gelatin from Marine Sources

Fish gelatin typically has a lower bloom value ranging from 0–270 g as compared to the bloom values for mammalian gelatin (130–308 g). Marine gelatins might exhibit a wider range of bloom values due to the differences in proline and hydroxyproline content in collagens from different types of species and habitat temperature. The range of viscosity values (cP) reported for gelatin skin of different freshwater fish species are from 1.87 to 3.63 cP [46]. Variation in viscosity value may be due to different fish species, environment and extraction method used. Generally, fish gelatins have lower concentrations of imino acids (proline and hydroxyproline) as compared to mammalian gelatins. A study by Ninan et al. [36] revealed that warm-water fish gelatins such as from bigeye-tuna and tilapia species have a higher imino acid content as compared to cold-water fish gelatin like from cod, whiting and halibut species. Muyonga et al. [47] reported that the proline and hydroxyproline levels for warm-water fish and cold-water fish were approximately 22–25% and 17%, respectively. Based on a study conducted by Sila et al. [43], the amino acid profile of the gelatin prepared from European eel (*Anguilla anguilla*) skin had a high proportion of glycine and imino acid residues. Overall, fish gelatin does exhibit good properties in films, remaining transparent, almost colorless, water soluble and highly extensible [48]. Numerous studies have been done on active and intelligent marine gelatin films such as active fish skin gelatin/peppermint essential oils composite films [49] and intelligent fish gelatin/haskap berries extracts composite films [50].

### 2.3. Gelatin from Poultry Sources

New gelatin sources such as poultry skin, feet, and bone have attracted attention as a substitution to mammalian resources [14,44]. Poultry species used include species from duck, chicken, and turkey. Avian gelatin was reported to possess amino acids, secondary structure, and molecular weight (285,000 g/mol) that nearly similar with mammalian gelatin (350.00 g/mol) [14,45]. Sarbon et al. [14] and Rahman and Jamalulail [51] reported that gel from chicken skin and chicken feet gelatin has a significantly higher bloom value (355.00 g and 264.33 g, respectively) as compared to bovine gelatin (229.00 g). Meanwhile, Nik Aisyah et al. [52] reported that duck feet gelatin with various acids treatment had higher bloom strength (225.53–334.17 g) than commercial bovine gelatin, which exhibited bloom value at 216.63 g. High bloom strength contributes to high melting temperatures and higher viscosity due to higher proportion of cross-linked component of ß and α chain. Sarbon [53] found that chicken gelatin exhibited a higher viscosity value at 150 mL/g as compared to bovine gelatin that was 127 mL/g. Furthermore, it was also found that chicken skin and duck feet gelatin possessed amino acids such as glycine (33.70 and 32.84%, respectively), proline (13.42 and 12.09%, respectively), hydroxyproline (12.13 and 9.65%, respectively) and alanine (10.08 and 11.06%, respectively) contributing to the higher gel strength and stability [14,54]. In addition, the imino acids (proline and hydroxyproline) value of chicken skin gelatin (25.55%) and duck feet gelatin (21.74%) were reported to be higher than that of bovine gelatin (23.33%) [14,54]. Gelatin from poultry sources exhibit good film forming properties as they showed high bloom value with high imino acids content [14,52]. Several studies that have been conducted on poultry-based gelatin films, including active chicken skin gelatin/Centella asiatica composite films [16,17] and active duck feet gelatin/cinnamon leaf oil composite films [54].

## 3. Film Forming Properties of Gelatin

Gelatin has been widely studied for its film-forming ability, especially in film production due to its outstanding filmogenic properties. Furthermore, it can be produced at a low cost while offering its unique properties as outer film to secure food from moisture loss and exposure towards light and oxygen [55]. Gelatins also have the ability to form physical gels and thermo-reversible gels. A gelatin gel formation is obtained from structural re-arrangement of protein formed by breaking the triple helix structures of collagen into single-strand molecules [56]. Gelatin based film are highly affected by its rheological properties, which depend on viscoelasticity, viscosity and processing temperature of the film. Gelation properties are mainly influenced by the shear storage modulus (G′), loss modulus (G″) and loss factor (tan δ), which are often measured as a function of time, strain and frequency. A gelling material can exhibit quite solid-like texture (G′ > G″) at a high frequency/fast timescale, but behaves much more liquid-like (G″ > G′) at low frequency/long time scales [57,58,59].

Gelatin viscoelastic properties are influenced by the amino acid composition, which is further affected by imino acid content and degree of prohydroxylation. Aside from that, average molecular weight distribution of different collagenous components (α- chains, β- or γ-components) and the ratio between α1- and α2-chains are also considered as important factor in determining the gelling properties [60]. It has been revealed that gelatin with higher content in α1-chains has exhibit higher viscoelastic properties, as α1-chains have better ability to refold than α2-chains. Additionally, higher levels of β-components have also been reported to contribute towards good gelling properties and promotes strength in the corresponding films as they stimulate better ability of renaturation to the fully collagen native form [60,61,62].

The physical properties of gelatin films depend on the characterization of raw materials and the extraction method used which derived from the different processing conditions and animal species. Furthermore, gelatin properties are also affected by physical parameters involved in film processing, such as the addition of substances or ingredients into the film processing method, including the inclusion of plasticizers [63,64], polymers [8,65,66] and cross-linkers [11]. Biodegradable films can be developed through casting or extrusion. Commonly, the casting method has been extensively reported in film formation process. This method involves the dissolving of biopolymer which then incorporated with either plasticizers or additives to obtain a film-forming solution. Later, the film-forming solution is cast onto plates and the solution is dried off [67,68].

Recent research showed that gelatin as food packaging has been making improvements in various types of food products such as fruits (strawberry, fresh apples cut, banana), fresh vegetables (tomatoes), meat (beef tallow, beef meat), marine products (fried salmon skin, fish sausage, chilled seabass fish fillets, Nile tilapia fish fillets, smoothhound breaded fish fillet, hake medallions, grass carp fish fillets), cheese, flax-seed oil, and drinks (red Fuji apple juice) [19,69]. Gelatin as an additional protective layer for fresh products has been proved to increase the shelf life of the intended food products by delaying microbial spoilage and providing moisture and gas barrier properties. Concurrently, fruits and vegetables that are coated with edible films containing active agents have longer shelf lives and their ripening processes are delayed [69].

## 4. Physical Properties of Gelatin-Based Film

### 4.1. Thickness of Gelatin-Based Film

Differences in film thickness might be influenced by the variability in nature, composition and solid content that present in the film structure [70]. The thickness in single bovine and porcine gelatin-based film were found in the range of 0.04–0.06 mm [18,39,71] and 0.07–0.11 mm, respectively [72,73,74]. Single porcine gelatin films have higher thickness value as compared to single bovine gelatin films. This is due to higher protein content in porcine gelatin (91.30%) as compared to bovine gelatin (88.45–91.20%) [36,75]. The higher constituents of protein concentrations in film formulation have induced an increase in solids content in the polymer matrix which in consequence will enhance the thickness of the film [76]. Meanwhile, in comparison to thickness value of single mammalian gelatin films, higher thickness value was observed in active mammalian composite gelatin films with incorporation of natural extracts such as oregano and lavender essential oils (0.07–0.15 mm) [38,77]. Additionally, higher thickness values were also observed in intelligent mammalian composite gelatin films in the range of 0.04–0.14 mm [14,39,71,72]. The higher thickness values observed in active and intelligent gelatin composite films are prompted by the increasing solids content in polymer matrix such as palmitic acid, linoleic acid, carvacrol, thymol and oleic acid compounds present in added natural extracts, which consequently enhances the film thickness layer.

In comparison with mammalian gelatin film, the thickness for single marine-based gelatin films derived from variety of sources has been reported in the same range (0.05–0.12 mm) as the thickness for single mammalian gelatin films [49,50,78,79,80,81]. This might be due to the similarly high levels of protein in both marine and mammalian sources. Marine gelatin derived from various types of fish species have been reported to exhibit 69.93–91.33% protein content [46,82,83]. While, the thickness for active marine gelatin composite film incorporated various types of natural extracts such as grape seed, basil, cinnamon and lavender essential oils were reported in higher value (0.06–0.21 mm) as compared to single marine gelatin films [26,79,80,81,84,85]. The higher thickness perceived for active marine’s gelatin composite films may be attributed to increased solid contents such as linalool, thymol, citral and safrole compounds, as well as presence of hydrophobic and volatility nature in natural extracts. The increment of these solid contents reduces the compactness of films and promotes higher density of structure. As for intelligent marine gelatin film, the thickness value of fish gelatin with incorporation of haskap berries extracts were observed at 0.05 mm with no significant difference as compared to single fish gelatin film [50]. This was due to the low amount of extracts has been incorporated into the film-forming solutions. Furthermore, the extracts were uniformly distributed within the gelatin polymer matrix which contribute towards non-significant thickness value between the single and intelligent composite gelatin film.

Moreover, in comparison to mammalian and marine gelatin film, single poultry-based film derived from chicken feet gelatin film has recorded low thickness value (0.06 mm) that was nearly similar to those thickness value reported for single bovine gelatin films [86]. The similar film thickness layer between single chicken feet and bovine gelatin films might be due to equivalent protein content in both gelatins that were reported at 88.35–90.06% and 88.45–91.20%, respectively [36,75,87]. Meanwhile, chicken feet composite film incorporated with sugarcane bagasse has obtained higher thickness values (0.07–0.09 mm) as compared to single chicken feet gelatin films [86]. The higher thickness obtained by chicken feet composite film were due to incorporation sugarcane bagasse that well-dispersed in the polymer matrix which increased the total solids content in the polymer matrix and enhance the thickness of the films. However, there has yet to be a study on thickness value for active and intelligent poultry gelatin film. Thus, based on these findings, it can be stated that film thickness value may vary according to the gelatin types, inclusion of other components into the films such as types of added natural extracts as well as the methods involved during the development process. The summaries of thickness for gelatin-based film from different types of gelatin sources are presented in Table 1, Table 2 and Table 3.

### 4.2. Color of Gelatin-Based Film

Generally, gelatin color depends on raw material used and extraction condition [113]. The color of food products was commonly measured in L*a*b* color parameters which indicated lightness, redness/greenness and yellowness/blueness axis, respectively. The color of film is an indicator for consumer acceptability, quality attributes and marketability. Normally, transparent film packaging has higher demands in market as customer would like to have clear view on the colors, textures and quality of the food’s ingredients that they are consuming. Higher transparent film packaging also indicated fresh and clean appearance with assumption these types of packaging might does not contain any unnatural ingredients or additives.

The L*, a* and b* color value reported for single mammalian-based gelatin film derived from bovine sources were found in the range of 89.07–97.30, −1.27–0.07 and 2.00–5.40, respectively while L*, a* and b* color value for single gelatin film from porcine sources were reported within the range of 90.00–96.97, −0.39–1.11 and 2.22–3.22, respectively [18,25,39,71,77,91]. The single porcine gelatin films exhibited more reddish color and less yellowish as compared to single bovine gelatin films. The reddish color might be attributed to red hue color possessed by glutamine and aspartate amino acids [114]. Porcine gelatins were reported to exhibit higher content of glutamine and aspartate amino acids (124.00 and 41.00 residues per 1000 amino acid residues, respectively) as compared to bovine gelatin which possessed glutamine and aspartate amino acids at 51.00 and 17.00 residues per 1000 amino acid residues, respectively [30]. Meanwhile, more yellowish coloration was observed in active mammalian composite film with incorporation of essential oils as compared to single mammalian gelatin films owing to their natural coloring components. The color value for active composite mammalian gelatin film incorporated with varied essential oils such as oregano, lavender, eugenol and ginger were ranged between L* (88.02–92.40), a* (−0.60–3.20) and b* (2.02–17.00), respectively [77,91]. The addition of essential oils has affected the film’s color due to its natural color pigment such as carotenoids and chlorophyll. In comparison to active mammalian gelatin films, the L*, a* and b* color value reported for intelligent mammalian gelatin film were found within the range of 28.00–91.53, −5.73–58.34 and −47.01–86.40, respectively [18,39,71,72]. The variation in s+ intelligent film’s color was attributed to the coloring components present in each added indicator. 

In comparison with mammalian gelatin films, the L*, a* and b* color values for single marine-based gelatin films derived from variety of sources such as tilapia, unicorn leatherjacket and catfish have been reported to range in between 90.32–94.25, −2.51–(−0.80) and −1.68–15.81, respectively [50,80,98,99,100]. Single marine gelatin films were observed to perceived more yellowish color as compared to single mammalian gelatin films. This might be attributed to higher content of cysteine and methionine amino acids in marine gelatin (1.00 and 10.00–17.00 residues per 1000 residues, respectively) as compared to mammalian gelatin [60,115]. Cysteine and methionine amino acids have been reported to rendered yellow spectral color [114]. While, active marine gelatin films were reported to have darker and more reddish color as compared to single marine gelatin films. The L*, a* and b* color values for active composite marine-based gelatin films with addition of natural extracts such as bergamot, lemongrass, epigallocatechin gallate, ginger, turmeric, plai, *Ziziphora clinopodioides* and grape seed were in the range of 55.12–91.11, −3.02–16.77 and 1.79–13.43, respectively [80,97,99,100]. The differences color in active composite film were due to the presence of pigment inherent in polyphenol, carotenoids and chlorophyll compounds that present in natural extracts which also affected by development of internal structure during drying process of film [80]. While, intelligent gelatin composite films have recorded as darker and reddish color as compared to single gelatin film. The L*, a* and b* color value for intelligent marine’s gelatin film has been observed at 75.55–90.35, 1.32–9.09 and −4.47–(−1.48), respectively [50]. The differences in intelligent film’s color were attributed to the natural coloring components present in the added extracts.

On top of that, single poultry gelatin films perceived less yellowish in comparison to mammalian and marine gelatin film. The L*, a* and b* value for single poultry-based gelatin film derived from chicken feet were reported in the range of 90.77–91.29, −1.40–(−1.30) and 3.18–3.01, respectively [86]. This might be attributed to lower content of cysteine and methionine amino acids in poultry gelatin (0.16 and 0.07%, respectively) as compared to marine and mammalian gelatin [14,60,115], where these two amino acids have been reported to rendered yellow spectral color [114]. However, there has yet to be a study conducted on color value for active and intelligent poultry gelatin film. Therefore, it can conclude that gelatin color was influenced by different types of raw materials, inclusion of other substances as well as the methods involved during the development process and it does not affect the nature and chemical quality of gelatin [116]. The summary of color properties for gelatin-based film from different types of gelatin sources are presented in Table 1, Table 2 and Table 3.

### 4.3. Light Transmission and Transparency of Gelatin-Based Film

Identification of organic compounds such as amino acids in gelatin-based film can be tested by using UV-vis spectrophotometer which uses visible light and ultraviolet in order to analyze and determine the chemical structure of substance. Commonly, a spectrophotometer is used to characterize electromagnetic radiation wavelengths in the range of 200–800 nm. As for gelatin films, being a good barrier to UV and visible light is indicated in the range of 200–280 nm and 380–800 nm, respectively. The transparency value of gelatin film has been regularly studied and calculated based on light transmittance at 600 nm. The summary of UV-Vis absorption spectra properties for gelatin-based film from different types of gelatin sources are presented in Table 1, Table 2 and Table 3.

#### 4.3.1. Ultraviolet Light Transmission of Gelatin-Based Film

Films with low UV light transmission value are a better barrier to UV penetration through the film. Several studies have reported on UV light transmission at 200–280 nm for single mammalian gelatin-based film derived from bovine sources detected between 0.00 to 24.41 [38,93]. Mammalian gelatin molecules exhibited aromatic amino acids such as phenylalanine, tyrosine, and tryptophan, which are known as sensitive chromophores that absorb light at wavelengths below 300 nm [66]. These amino acids have conjugated pi bonds from aromaticity in which the entire structure acts as a chromophore. Conjugated bonds hold two electron pairs in which each electron possesses an independent and opposite spin of equal energy. When a photon of ultraviolet radiation energy strikes an electron, it is induced to rise to an excited (higher energy) level and is able to travel the entire organic structure without breaking the bonds [117]. Composite films of bovine gelatin/carboxymethyl cellulose (CMC) have also been reported to exhibit low UV light absorption within the range of 0.15–4.00 [63,66]. These findings indicate that gelatin proteins from bovine exhibited good UV-barrier properties due to the presence of aromatic amino acids that absorb UV light. Meanwhile, intelligent bovine gelatin film with curcumin incorporation showed an increase of absorption peaks in the UV region as compared to the single bovine gelatin film. This phenomenon is attributed to curcumin degradation products under alkaline conditions such as trans-6(4′-hydroxy-3′-methoxyphenyl)-2,4-dioxo-5-hexanal, ferulic aldehyde, ferulic acid, feruloylmethane and vanillin [39].

Meanwhile, studies on light transmission at UV ranged (200 to 280 nm) for single marine-based gelatin film derived from various fish species showed higher values (0.01–40.73) as compared to the bovine gelatin-based film [79,93,100,102]. Higher absorption of UV light transmission might be attributed to lower aromatic amino acids such as phenylalanine (1.3–18.27%) and tyrosine (0.3–5.42%) that present in fish gelatin as compared to mammalian gelatin which were reported to exhibit phenylalanine and tyrosine in the ranges of 1.60–27.00% and 1.16–26.00%, respectively [14,30,82,118]. The result for active composite fish gelatin-based film with addition of natural extracts such as bergamot, ginger, turmeric, plai, kaffir lime, lemon, lime and green tea were reported to have lower UV light transmission (0.00–26.01) [79,100,101,102]. The results showed that film incorporated with natural extracts has good ability in preventing UV transmission as the results revealed low value of absorbance (0.00–26.01) [79,100,101,102]. The lower absorbance might be attributed to light scattering of natural extracts droplets which disperse in the film matrix and obstruct the transmission of light [100,101]. Meanwhile, intelligent fish gelatin/haskap berries extract composite film has recorded lower absorption peaks in the UV region as compared to the single fish gelatin film due to UV–vis absorption ability by aromatic rings of polyphenols presents in the added extracts [50].

Next, single poultry gelatin-based films derived from chicken skin have been reported to have lower UV light transmission (0.03–4.48) as compared to the mammalian and marine gelatin-based film [8,64,66]. Nazmi et al. [66] stated that the UV range for chicken skin gelatin films blended with CMC was detected at 2.07–4.00, indicating lower UV light transmission when compared to single chicken skin gelatin film (2.59–2.93). This is due to the formation of intermolecular bonding between gelatin and CMC being able to prevent the penetration of UV light into the film. Meanwhile, active chicken skin gelatin-based film with incorporation of Centella asiatica has recorded lower UV light transmission (0.00–0.02) in comparison to single chicken skin gelatin film (0.00–0.42). The lower transmission value may be attributed to the obstruction of light due to the light scattering effect originated from uniform dispersion of the natural extracts in the film matrix. However, there has yet to be a study conducted on UV light transmission for intelligent poultry gelatin film. This indicates that chicken skin gelatin film may be able to prevent UV transmission, as these poultry gelatins exhibit aromatic amino acids such as tyrosine, phenylalanine and tryptophan, which offer good UV barrier properties [66].

#### 4.3.2. Visible Light Transmission of Gelatin-Based Film 

The light transmission value for single mammalian gelatin film derived from bovine hide and skin were reported in the range of 63.90 to 88.76 [38,93]. Meanwhile, findings by Nazmi et al. [66] revealed that bovine gelatin with addition of CMC blended film exhibited lower visible light transmission value (0.82–0.65) as compared to single mammalian gelatin films. Similarly, incorporation of N-chitin and corn oil into active bovine gelatin-based film has also been reported to exhibit lower visible light transmission value (1.47–4.05) as compared to single bovine gelatin films (3.61–5.44) [38]. The single gelatin films were observed to transmit significant amounts of visible light. The incorporation of additional substances such as corn oil has seemed to reduce exposure to visible light by scattering which might be attributed to C=O bond in the polymer matrix and able to help in blocking a particularly damaging range of wavelengths. Similar findings were found by Iahnke et al. [90] which observed lower absorption value in active bovine gelatin film with incorporation of carrot residue fiber (0.03–58.91) as compared to the single bovine gelatin film (36.50–83.00). This phenomenon may be attributed to the presence of carotenoids in carrot fiber that have specific absorption in the blue region of the spectrum. As for intelligent bovine gelatin film, the gelatin-curcumin films at pH 6 and pH 11 had absorption peak at 420 nm and 460 nm, respectively owing to their red color properties [39]. Thus, it can be seen that bovine composite films have better visible light barrier properties and are able to achieve lower oxidation rates as compared to single bovine gelatin-based film.

Meanwhile, in comparison to mammalian gelatins, single marine gelatin-based films derived from various fish species have been reported to exhibit lower light transmission within the range of 28.36–90.50 [3,79,93,101,102,110]. The major influence for absorption of light transmission in this wavelength range could be attributed to higher double bonds structure that present in some amino acid compounds of gelatin such as glutamine, tyrosine and phenylalanine. The compounds with higher double bonds might have lower light transmission value as double bond’s structure are responsible for the absorption of visible radiation [119]. Marine gelatins have been reported to exhibit higher composition of amino acids that have a double bonds structure such as glutamine (6.14–6.58%), tyrosine (1.84–5.42%) and phenylalanine (16.10–18.27%) in comparison to bovine gelatin, which was reported to exhibit glutamine, tyrosine and phenylalanine at 5.43%, 1.16% and 1.60%, respectively [14,118,120]. Meanwhile, visible light transmission value for active marine gelatin composite film incorporated with natural extracts such as bergamot, lemongrass, grape seed, kaffir lime, lemon, lime, ginger, turmeric, plai, and green tea were in accordance with the findings for mammalian composite films which showed lower light transmission value (6.20–88.23) as compared to single gelatin films [97,100,101,102]. The decreasing value of light transmission at visible range might be attributed to light scattering of natural extracts droplets distributed throughout the protein network as well the interaction formed between natural extracts and gelatin polymer [97,100]. As for intelligent fish gelatin/haskap berries extract composite film, the lower absorption peaks in the visible regions were observed as compared to the single fish gelatin film [50]. This might be attributed to the addition of natural extracts which exhibited phenolic compounds and unsaturated bonds that absorb the visible light.

In comparison to mammalian and marine gelatin film, the light transmission single for poultry gelatin-based film in visible range were detected at lower value (3.98–87.58) [8,64,66,86]. This might be due to higher composition of amino acids that have a double bonds structure such as glutamine (5.84%), tyrosine (1.22%) and phenylalanine (1.77%) in chicken skin gelatin as compared to bovine gelatin [14]. The compounds with higher double bonds structure might have lower light transmission values, as these double bonds are responsible for the absorption of visible radiation [119]. While, the light transmission for chicken skin gelatin/CMC and active chicken skin gelatin/CMC/Centella asiatica composite film had resulted in lower value of 0.61–1.08 and 0.03–9.24, respectively as compared to single chicken skin gelatin film (3.98–11.98) [16,66]. The lower values obtained were governed by the alignment or arrangement of polymer in film, which are able to obstruct the transmission of visible light. Thus, chicken skin gelatin composite film exhibited better barrier of visible light penetration through the film as it obtained lower light transmission. However, there has yet to be a study conducted on visible light transmission for intelligent poultry gelatin film.

#### 4.3.3. Light Transparency of Gelatin-Based Film 

Higher transparency values indicate high film opacity [16]. A film’s transparency might be influenced by variation of composition, density and the structure of aggregation or alignment in gelatin molecules that modifies the refractive index and restrict the light passage through the film matrix [94]. The single mammalian gelatin films derived from porcine and bovine sources had been reported to exhibit light transparency value between the range of 0.35–0.63% and 0.59–2.43%, respectively [38,63,73,93]. Active bovine gelatin composite film incorporated with different concentration of corn oil have been found to exhibit higher transparency value (5.38–5.94%) which indicates more opaque appearance than reported value for single bovine gelatin film [38]. The results were in line with finding by Gómez-Estaca et al. [121] on higher opacity index in bovine-hide composite films with incorporation of oregano (0.542–0.725%) and rosemary extracts (0.530–0.684%) as compared to single bovine-hide gelatin film (0.461%). This shows that the inclusion of hydrophobic substances such as corn oil and natural extracts could increase the opacity of a film due to its coloring components and a reduction in the ordered film protein network [38,100]. However, there is still no study reported for light transparency value of intelligent mammalian gelatin-based film.

In comparison to mammalian gelatin films, single marine gelatin-based films derived from varied fish species showed similar light transparency values (0.05 to 2.86%) [79,100,101,102,110]. This might be due to similar microstructure in single marine gelatin films that exhibited smooth and homogeneous surfaces without grainy and porous structures as single mammalian gelatin films [73,100,101]. However, the active marine gelatin composite films incorporated with different types of natural extracts such as bergamot, kaffir lime, lemon, lime, ginger, turmeric, plai and green tea resulted in higher transparency value (1.12–5.66%) as compared to single marine gelatin films [79,100,101,102]. A higher transparency value indicates high opacity of film. Based on the studies conducted, it can be stated that the addition of natural extract was found to decrease the transparency of the film. This phenomenon might be attributed to natural coloring components present in each extracts along with different structure of aggregation or alignment in gelatin molecules evolved from the reaction with natural extracts compound [100]. In addition, the inclusion of natural extracts such as essential oils have increased the intensity of light scattering in film matrix due to its hydrophobic nature and formed crosslinking with the gelatin polymer [122]. These factors have contributed to the compactness of film matrix and decreased the films transparency. However, there is still no study on light transparency has been conducted for intelligent marine’s gelatin film.

Lower transparency value has been perceived in single poultry gelatin-based film as compared to single mammalian and marine gelatin-based film. The transparency value for chicken skin and feet gelatin-based film were observed within the range of 0.77–1.94% [86]. The lower value was attributed to smaller, compact and more organized microstructure possessed by poultry gelatin film which increased the films opaqueness and lowering the amount of light passes through the film as compared to the others gelatin film sources [94]. Meanwhile, chicken skin gelatin composite films have been reported to exhibit higher transparency value (0.71–3.97%) as compared to single chicken skin gelatin films (0.77–1.94%) [8,16,64]. The greater transparency value represents lower transparency of film [64]. The higher transparency value obtained in chicken skin gelatin composite films were due to the incorporation of additional substances that formed crosslinking with gelatin polymer and produced more compact film matrix which subsequently increased the film’s opacity [8]. Chicken skin gelatin composite films resulted in lower transparence and could be as excellent barrier to prevent light penetration while inhibiting lipid oxidation from occurring in foods [64]. Meanwhile, there has yet to be a study conducted on light transparency for intelligent poultry gelatin-based film.

### 4.4. Water Vapor Permeability (WVP) of Gelatin-Based Film

A study on WVP for single mammalian gelatin-based film derived from bovine sources reported a range between 8.00 × 10^−11^–9.68 × 10^−10^ g/m·s·Pa [38,71,77,93]. Meanwhile, active bovine gelatin/N-chitin composite film (8.89 × 10^−10^ g/m·s·Pa) has been reported to offer lower WVP values as compared to the single bovine gelatin film [38]. The incorporation of chitin nanoparticles helps in decreasing film’s WVP by creating a tortuous pass way across the film, increased polymer cohesiveness by filling free volume spaces between polymer chains and lowered free volume for moisture transmission [38]. In addition, the incorporation of corn oils into active bovine gelatin-based films showed lower WVP values (0.68–7.86 × 10^−10^ g/m·s·Pa) as compared to single bovine gelatin films. The incorporation of corn oils was revealed to increase the hydrophobic phase of polymer and reduce the film’s tendency toward water uptakes capacity [38,77]. As for intelligent mammalian gelatin-based films, the WVP values for bovine gelatin composite films incorporated with color indicator have been reported in a range of 6.50 × 10^−11^–1.2 × 10^−10^ g/m·s·Pa [18,39,123]. The inclusion of color indicator such as curcumin extract has been found to yield better water vapor properties as compared to single bovine films [39]. The lower WVP value might be due to the hydrophobic structure composed of long carbon chain and benzene ring with prolonged tortuous pathways owned by curcumin. These structures help to restrain or prolong the penetrating path of water vapor through the films, leading to a more cohesive structure and lower free volume for moisture transmission [124].

In comparison, single fish gelatin films have been reported to exhibit lower WVP values as compared to single bovine gelatin films [40,125]. The statement supported with the findings by Gómez-Guillén et al. [103], Hosseini et al. [78], Jridi et al. [79] and Tongnuanchan et al. [81] which found that single films from various types of fish species showed lower WVP value (6.00 × 10^−13^–2.05 × 10^−10^ g/m·s·Pa) as compared to values reported for bovine gelatin film. The lower WVP values of single fish gelatin film from several species can be explained in terms of the amino acid composition; the fish gelatins are comprised of higher constituents of hydrophobicity due to lower proline and hydroxyproline contents. The hydroxyl group of hydroxyproline is normally available to form hydrogen bonds with water [126]. The incorporation of natural extracts such as leaf extract, ginger, turmeric, plai, bergamot, kaffir lime, lemon and lime essential oils into active fish gelatin-based film has been found to improve the water barrier properties of the films (7.97 × 10^−13^–1.90 × 10^−10^ g/m·s·Pa) as compared to single fish gelatin-based film [78,79,100,101,103]. These occurrences were due to the hydrophobic nature exhibited by the essential oil which consequently increase the hydrophobicity of films and thus reduced the water vapor migration through the film [100,101]. In addition, some natural extracts are able to reduce WVP by forming more compact structure with polymer matrix derived from internal crosslinking. As for intelligent marine gelatin film, the WVP value for fish gelatin film with incorporation of haskap berries extract has recorded lower value (5.96–7.14 × 10^−10^ g/m·s·Pa) as compared to single fish gelatin film (8.33 × 10^−10^ g/m·s·Pa) [50]. This was attributed to more compact network and decreased amorphous regions of film matrix in intelligent fish gelatin composite films which reduced the diffusion of water vapor through the films.

As for poultry gelatin film, studies by Nazmi et al. [66], Nor et al. [64], Soo and Sarbon [8] and Tew et al. [86] showed that WVP for single chicken feet and chicken skin gelatin film have been observed to range in lower value (4.17 × 10^−12^–5.94 × 10^−10^ g/m·s·Pa) as compared to mammalian gelatin films. Although chicken skin gelatin is perceived to have a higher content of imino acid (proline and hydroxyproline) available to form hydrogen bonds with water as compared to bovine gelatin, they also possessed higher content of certain non-polar amino acid such as proline (13.42%), alanine (10.08%), leucine (2.63%), isoleucine (1.15%), phenylalanine (1.77%) and tyrosine (1.22%) in comparison to bovine gelatin [14,74]. These non-polar amino acids are associated with higher hydrophobicity of the polymer network, and are thus prone to reduce the permeability of water vapor through the films. Meanwhile, chicken skin gelatin composite film incorporate with rice flour showed that WVP values of the films were significantly increased (*p* < 0.05) from 6.83 × 10^−10^ to 1.39 × 10^−9^ g/m·s·Pa with the increased concentrations of rice flour [8]. This was attributed to hydrophilic nature of rice flour, which promotes greater water affinity and enhanced the migration of water vapor molecules through the gelatin films [8]. In addition, a study by Nazmi and Sarbon [16] mentioned that active chicken skin gelatin/CMC composite films with incorporation of Centella asiatica extract showed higher WVP values (1.11–1.13 × 10^−4^ g/m·s·Pa) as compared to control chicken skin gelatin/CMC composite films (1.03 × 10^−4^ g/m·s·Pa). The addition of Centella asiatica extract was reported to have no significant effect on cross-linking in film polymer matrix and exert no influence in WVP. The increased values for water permeability were caused by higher degrees of hydrogen bonding derived from highly polar polymers in active chicken skin gelatin composite films [16]. However, there has yet to be a study conducted on WVP for intelligent poultry gelatin film. Thus, based on these findings, it can be stated that the WVP value may be varied according to the gelatin types, extraction method and inclusion of other components into the films. The summary of WVP properties for gelatin-based film from different types of gelatin sources are presented in Table 1, Table 2 and Table 3.

### 4.5. Melting Point (T_m_) of Gelatin-Based Film

The melting point (T_m_) of single mammalian gelatin-based films derived from bovine and porcine were reported within the range of 60.42–82.20 °C and 66.80–87.70 °C, respectively [38,94,95,96]. A slightly higher melting point observed in porcine gelatin films were prompted by higher imino acid composition (23.70%) in comparison with bovine gelatin films (22.91–23.33%) [14,74]. It is known that higher imino acid content will increase the coil–helix transition temperature as it has direct correlation to the thermal stability of protein via hydrogen bond [3,81]. Meanwhile, the melting point (T_m_) of active bovine gelatin-based nanocomposite films incorporated with N-chitin was detected at higher T_m_ value (102.9 °C) as compared to the single bovine gelatin films [38]. The higher melting point in active bovine gelatin-based nanocomposite films might be due to the incorporation of N-chitin, which helps in increasing the overall crystallinity of polymer which leads to higher transition temperature and enthalpy [109]. In addition, active bovine/corn oil composite films also have resulted in wider range of melting temperature (104.50–113.00 °C) as compared to the reported single bovine gelatin films [38]. The wider range might be attributed to the overlapping melting peaks of lipid phase and gelatin or decreasing crystallinity of the films that consequently led towards increasing amorphous phase. As for intelligent furcellaran/porcine gelatin composite films with incorporation of green tea and pu-erh extracts, the T_m_ values were observed at lower value that ranged between 161.80–174.10 °C and 158.40–168.90 °C, respectively as compared to control furcellaran/porcine gelatin composite films (173.50 °C) [72]. The lower value might be attributed to the addition of extracts which exhibited hydrophobic nature and caused discontinuity in the macromolecular network in the film matrix, thus lowering the required energy for disruption [105].

While, the melting point for single marine gelatin-based film derived from various types of fish skin were reported to exhibit lower value of endothermic melting transition (T_m_) (53.14–124.45 °C) as compared to single mammalian gelatin films [3,81,105,111]. This is due to lower imino acid composition present in marine gelatin such as big eye snapper, rohu and carp skin gelatin (14.43–20.86%) as compared to mammalian gelatin [74,127]. Meanwhile, the fish skin gelatin composite film with incorporation of palm oil, basil essential oils, citronella essential oils and squalene rich fraction from spot-tail shark liver have resulted the T_max_ value within the range of 107.60–123.52 °C, 74.83–77.92 °C, 77.58–83.08 °C and 110.08–123.79 °C, respectively [81,104,105]. The addition of palm oil into the gelatin film has higher T_max_ value as compared to the single gelatin films which might be associated with the lower water content in film [81]. The addition of natural extracts such as essential oils into the films requires a higher enthalpy for disruption of the interchain interaction and thus results in higher melting point as compared to single gelatin films. This might be due the formation of crosslinking such as hydrogen bonds or hydrophobic interactions between natural extracts with the reactive groups of polypeptides in gelatin. However, there has yet to be a study conducted on melting point for intelligent marine’s gelatin film.

As for single poultry-based gelatin film derived from chicken skin, the melting points (T_m_) was reported in higher value within the range of 49.51–134.22 °C as compared to single bovine and marine gelatin films [8,66,94]. This was due to higher composition of imino acid (25.55%) that would have contributed to a stiffer and more rigid gelatin structure [14]. Hence, this required higher energy and time needed for the helix–random transition of gelatin and breakage of hydrogen bonds between the polypeptide chains in gelatin molecules [8,81]. The T_m_ for chicken skin gelatin composite films with incorporation of CMC was reported at lower value (126.93 °C) as compared to single chicken skin gelatin film (134.22 °C) due to the evolution of residual water [66]. In addition, Soo and Sarbon [8] reported that chicken skin gelatin/rice flour composite films exhibited higher T_m_ value with two separated endothermic peaks as compared to single chicken skin gelatin film (49.51 °C). Meanwhile, active chicken skin gelatin/CMC composite film incorporated with Centella asiatica extract has resulted in higher T_m_ value (130.11–131.31 °C) in comparison to control chicken skin gelatin/CMC composite film (124.38 °C) [16]. The studies revealed that the addition of rice flour and Centella asiatica extract increased the T_m_ value due to crosslinking reaction between added substance with gelatin polymer matrix, which has caused a reduction in mobility of biopolymer chains and thus resulted in a higher melting point. However, there has yet to be a study conducted on melting point for intelligent poultry gelatin film. The summary of thermal properties for gelatin-based film from different types of gelatin sources is presented in Table 1, Table 2 and Table 3. 

## 5. Mechanical Properties of Gelatin-Based Film

### 5.1. Tensile Strength of Gelatin-Based Film

Tensile strength (TS) of packaging materials is important in determining the protection and tampering resistance of food packaging [128]. Higher tensile strength is generally preferred for a variety of packaging products as they ensure better seal with secure load stabilization while also contribute towards safer and high-quality products for the end consumer. 

Several studies on TS value for mammalian gelatin-based film have been reported. The TS value for single pig skin gelatin-based film were found in the range of 2.40–63.25 MPa [25,74,91,92] which were higher value as compared to the TS value of single bovine gelatin films (0.70–51.68 MPa) [71,74,77,94]. The higher TS value obtained were attributed from higher gel strength possessed by porcine gelatin that exhibited relatively high Bloom values (300 g) as compared to bovine gelatin (229 g) [14,74]. The mechanical properties of gelatin films are correlated with the triple-helix content. The higher gel strength possessed by porcine gelatin indicates more rigidity and a higher stiffness of the gelatin’s structure. Meanwhile, active pig skin gelatin composite films with incorporation of natural extracts such as eugenol, ginger, cinnamon, guarana, rosemary and boldo-do-chile were found to have higher TS values (3.20–35.00 MPa) as compared to single gelatin films [91,92]. Higher TS values in composite films might be due to the hydrophobic interaction between protein and polyphenol and also formation of hydrogen bonds that led to film strengthening. On the contrary, the TS value reported for active bovine gelatin composite film with incorporation of natural extracts from plants such as corn oil, oregano and lavender essential oils have recorded opposite findings, observing a lower TS value (2.70–39.47 MPa) as compared to reported TS for single gelatin films [38,77,88]. The reduction in TS value was due to discontinuities in overall polymeric structure caused by incompatible reaction between added extracts with film nature and later resulted in weakening the film’s mechanical strength [38]. As for intelligent bovine gelatin composite film, the TS values were ranged between 1.90–3.40 MPa [39]. The higher TS value in intelligent mammalian gelatin composite films as compared to single mammalian gelatin films might be prompted by good compatibility and higher formation of intermolecular hydrogen bond and hydrophobic interaction which stimulated greater TS value with stronger film’s structure. 

In comparison to mammalian gelatin films, TS value reported for single marine gelatin film from various types of fish species were found in lower value (6.23–43.62 MPa) [78,79,85,100]. The lower TS values were attributed to lower imino acids (proline + hydroxyproline) content in marine gelatin as compared to mammalian gelatin. Higher composition of imino acid (proline + hydroxyproline) would contribute to more stiff and rigid gelatin structure since triple helix of gelatin molecules is made up of repetitious amino acid sequence glycine–X–Y, where X and Y are mostly proline and hydroxyproline amino acids [129]. On top of that, the differences in TS value between each single fish gelatins films were due to different types and species of fish with different structural compounds, amino acid compositions and sizes of protein chains, resulting in different film formations [3]. In addition, the active fish gelatin composite film with incorporation of natural extracts such as origanum vulgare, ginger, turmeric, plai, basil and palm oil showed lower TS values (3.28–35.73 MPa) as compared to the control fish gelatin-based film [78,100,106]. The inclusion of hydrophobic substance such as natural extracts and essential oils into gelatin composite films generally showed a reduction of the TS value, as these substances may hinder or interfere with protein-protein interaction in the film network. This leads to the discontinuity of film matrix, lack of cohesive structure integrity of film network and thus, lowers the strength of films [81,100]. As for intelligent marine gelatin film, the TS value for fish gelatin film with incorporation of haskap berries extract has recorded a higher value (46.7–51.5 MPa) as compared to single fish gelatin film (42.5 MPa) [50]. The higher TS values were prompted by the formation of intermolecular hydrogen bonds formed between the added extracts with gelatin polymer matrix which has created more compact films with higher resistant to applied tensile stress [50].

Moreover, poultry gelatin film from single chicken feet and chicken skin gelatin-based film has been reported to exhibit higher TS value than reported marine gelatin films (34.20–44.86 MPa and 0.98–33.66 MPa, respectively) [8,64,66,86]. In addition, a study by Suderman et al. [94] found that single chicken skin gelatin films exhibited the highest TS value (5.57 MPa) compared to bovine (2.97 MPa) and porcine (3.21 MPa) gelatin films. The higher TS values were owing to higher imino acid content (25.55%) exhibited by chicken skin gelatin as compared to mammalian gelatin which were reported to yield within the range of 23.33–23.70% [14,74]. Meanwhile, the chicken skin gelatin composite film with CMC incorporated (5.53 MPa) has recorded higher TS value as compared to single chicken skin gelatin film due to the increased of film stiffness caused by incorporation of CMC [66]. A similar phenomenon was also obtained by Soo and Sarbon [8], who reported higher TS value for chicken skin gelatin composite film with addition of rice flour (2.08–2.91 MPa) attributed to the formation of hydrogen bond between rice flour with gelatin molecules. The TS value for active chicken skin gelatin/CMC composite film with incorporation of Centella asiatica extract was found to be in higher value (4.50–5.00 × 10^−2^ MPa) as compared to chicken skin gelatin/CMC composite film (3.00 × 10^−2^ MPa). The higher TS value in active chicken skin gelatin composite films were attributed to the formation of hydrophobic interaction and hydrogen bond between phenolic compounds of the added Centella asiatica extract with the gelatin molecules which strengthened the film matrix and increase the TS value [16]. However, no study has reported on the TS value of intelligent gelatin film from poultry. The summary of mechanical properties for gelatin-based film from different types of gelatin sources is presented in Table 1, Table 2 and Table 3.

### 5.2. Elongation at Break (EAB) of Gelatin-Based Film

Elongation at break is the ratio between changed length and initial length after breakage of the tested specimen [130]. It is related to the capability of plastic specimen to resist changes of shape without crack formation. The EAB value reported for single mammalian gelatin film derived from bovine and porcine were found in the range of 0.78–30.83% and 4.40–90.55%, respectively [38,71,74,92]. A higher EAB value perceived by single porcine gelatin films were owing to higher content of imino acids that indicates better viscoelastic properties and greater ability to develop a stronger gel structure [131]. Meanwhile, mammalian gelatin composite films with incorporation of polymer (tapioca starch, N-chitin), natural extracts (boldo, guarana, cinnamon, rosemary, eugenol, ginger, oregano, lavender, Zataria multiflora) and metal oxide (nanorod zinc oxide) have obtained higher EAB value (2.10–198.60%) [71,77,88,91,92]. The higher EAB value observed in gelatin composite film incorporated with hydrophobic substances such as natural extracts were reported to reduce the intermolecular forces between gelatin polymer chains and consequently promotes film flexibility, as well as the chain mobility [91,92]. In addition, intelligent bovine gelatin film with incorporation of curcumin a recorded lower EAB value (144.30–198.60%) as compared to single bovine gelatin film (159.20–206.90%) [39]. This might be attributed to poor interfacial interaction between the polymer chains and curcumin which consequently caused reduction in film extensibility.

In comparison to mammalian gelatin films, the EAB value for single marine gelatin film derived from various types of fish species were reported within the range of 2.96–76.73% [50,78,79,81,112]. The lower EAB values were prompted by lower imino acids content present in marine gelatin as compared to mammalian gelatins. Meanwhile, active marine gelatin film has observed higher EAB value (41.70–151.82%) as compared to single marine gelatin films [78,100,106]. Many studies found that active gelatin composite film with incorporation of essential oils or natural extracts such as bergamot, lemongrass, basil, cinnamon, peppermint, citronella and palm oil resulted in higher EAB value due to these compounds might exhibited plasticizing effect in resulting film which could increase the free volume between gelatin molecules and enhance greater mobility, thus resulting in higher extensibility of resulting films [106]. In addition, the EAB value for intelligent furcellaran/carp gelatin composite film with incorporation of rosemary extract and intelligent cold water fish skin gelatin film with incorporation of haskap berries extract were found at higher value that accounts for 69.63–75.71% and 2.87–3.69%, respectively as compared to control gelatin composite film [50,107]. The higher EAB value in intelligent gelatin films was owing to the formation of new interactions between phenolic compounds with the side chains of proteins or amino acids which led to more stretchable film’s structure [107]. Furthermore, polyphenols present in the added extracts could act as plasticizers, which enhanced the flexibility of the gelatin films [50]. 

Moreover, single poultry gelatin films from chicken feet and chicken skin have recorded higher EAB value (3.87–561.00%) as compared to single mammalian and marine gelatin films [8,64,66,86]. The higher EAB values were attributed to higher imino acid composition in chicken skin gelatin that accounts for 25.55% [14] compared to bovine and porcine gelatin which reported at 23.33 % [14] and 23.70% [74]. Higher composition of imino acids have promoted stronger triple helix formation and increase the viscoelasticity properties. Thus, it can be stated that chicken skin gelatin composite film has demonstrated greatest mechanical strength and extensibility properties in comparison with other films. In addition, a study by Soo and Sarbon [8] showed an increasing trend of EAB value (58.45–79.31%) for chicken skin gelatin film with increasing concentrations of rice flour. This was due to higher formation of strong hydrogen bond between the gelatin molecules with the available hydroxyl groups in rice flour. This interaction lowers the molecular mobility and results in higher chain entanglement and molecular slippage upon tensile deformation which increased the film’s extensibility [8]. The EAB value for active chicken skin gelatin/CMC composite film with incorporation of Centella asiatica extract was found to be higher (271.17–281.00%) as compared to chicken skin gelatin/CMC composite film (223.05%) [16]. This might be attributed to the plasticizing effect derived from polyphenols contents present in the extracts, which are able to enhance the flexibility of the gelatin films [50]. The mechanical properties for gelatin-based film from different types of gelatin sources are presented in Table 1, Table 2 and Table 3.

## 6. Biodegradability of Gelatin-Based Film

The degradation rate of a gelatin-based film is mainly dependent on its molecular weight. The polymers chains need to reach sufficiently low molecular weight in order to be metabolized by microorganisms. Subsequently, the microorganism will further convert the carbon in the polymer chains into carbon dioxide or assimilate it into biomolecules [132].

The biodegradation rate for single bovine gelatin films were reported within the range of 18.00–25.00% for 3 days’ observations [133,134,135]. High biodegradation rate observed in mammalian gelatin films might be due to the high level of hydroxyproline in mammalian gelatin. The hydroxyl group of hydroxyproline is normally available to form hydrogen bonds with water [126]. Thus, this has made the films become more susceptible towards microorganism attacks due high content of moisture. Meanwhile, bovine gelatin composite films with incorporation of additional substances such as dialdehyde starch, sodium montmorillonite, carboxymethyl cellulose (CMC) and sorbitol have recorded lower biodegradation rates that were observed at <10–20%, 20%, 6.03% and 4.16–6.20% as compared to single bovine gelatin films for 3 days’ observations [63,133,134]. The lower biodegradation rate in gelatin composite films might be influenced by the higher molecular weight, more compact structure and formation of covalent crosslinkages between the added substances and a gelatin polymer matrix [63,134]. These factors have caused slower extent of microbial attack and proteolytic enzyme reaction and thus, induced lower hydrolysis reaction which led to lower biodegradation rate [63]. Meanwhile, a study by Suderman et al. [89] has reported higher degradation rate for bovine gelatin/CMC/chitosan composite films (40.09–85.50%) as compared to single bovine gelatin films (48.81–55.29%) for 5 days’ observations. The higher degradation rate in bovine composite films may due to higher hydrophilic structure and heterogeneous mixture of water-soluble proteins that presence in film formulation with higher proportions of gelatin and chitosan. The higher hydroxyl components have exposed to the films towards higher susceptible extent of microbial attack and increase the hydrolysis reaction which consequently accelerated the film’s degradation rate. However, no study has reported on biodegradability rates for active and intelligent mammalian gelatin film.

In comparison to single mammalian gelatin films, the biodegradation rate for fish bone gelatin/chitosan/tapioca flour composite films has lower values, within the range of 8.48–11.15% during first day of observation [108]. This might be due to the low molecular weight of by fish gelatin as compared to mammalian gelatin [40], since the degradation rate of gelatin-based film is mainly dependent on its molecular weight. In addition, a study by Susilawati et al. [108] also found lower degradation rate in film with higher concentration of gelatin and chitosan. This was attributed to lower water content in composite films as higher concentration of chitosan was added which caused larger energy needed for microorganisms to breakdown the proteins in films [108]. However, no study has reported on the biodegradability rate for active and intelligent marines and poultry’s gelatin film as well as for single poultry’s gelatin films. Thus, it can be concluded that the biodegradability rate is mainly depends on the molecular weight and total solid content present in each film packaging. The summary of biodegradation properties for gelatin-based film from different types of gelatin sources is presented in Table 1, Table 2 and Table 3.

## 7. Current and Future Trends

Nowadays, many approaches and studies have been conducted on the development of gelatin film packaging utilized from alternative sources such as seafood and poultry in order to fulfill consumer needs and address the increasing global demand for gelatin. Furthermore, at the present times, many studies also have been done to improve and extend gelatin-based film properties and their functionality by incorporating various types of active substances such as natural extracts, essential oils, metal oxides, etc. The inclusion of these active substances has been found to enhance the microbiological safety and sensory properties, while also maintaining the quality of the intended products [107].

Future trends should focus more on toxicity, migration assays, and risk assessment involved when using the active or intelligent agents in gelatin packaging film as well as their potential impacts on human health and the environment. Furthermore, more research efforts are needed in the quest for materials that can improve the gelatin film barrier and functional properties including the integration of nano-engineered materials as these films are still far behind the excellent barrier function provided by synthetic packaging. Moreover, there is still lack of study done on the actual application of gelatin-based film in industrial scale. Thus, there should be more application tests done on gelatin-based film including the use of these films in packaging machinery and as flexible active and intelligent packaging for various food products that ease the consumer needs. There also should be more research done on labelling details with current technology, such as flexography printing for gelatin-based film.

## 8. Conclusions

Gelatin-based films are attracting great interest from researchers and food manufacturers all around the world, as they offer excellent characteristics through their mechanical and barrier properties and exhibit wide applications as biodegradable food packaging able to substitute for synthetic film packaging. Gelatin films in general have been widely reported as a good capping mediator for metallic ions and has a good matrix for these active agents to carry out their specific functions for enhancing the safety, stability, functionality, and shelf-life of food products. On top of that, current studies on gelatin thin films have demonstrated great potential in tissue engineering and clinical settings. The combination of these materials has proven its synergistic effects in wound-healing applications such as delivering drug to heal a wound in a rat model [136,137]. This is due to good film’s strength required for the wound-healing applications. Moreover, gelatin thin films also have been studied as a promising candidate for organic memory applications [138]. As for gelatin films properties, the difference of thickness, color, and biodegradability between all types of gelatin films were mainly attributed by the total solid content and incorporation of additional substances in each film. Meanwhile, for UV and visible light absorption, poultry gelatin films have recorded lower values compared to mammalian and marine gelatin films due to higher composition of amino acids that have a double bond structure such as glutamine, tyrosine, and phenylalanine. In addition, marine gelatin films have been observed to have lower WVP value compared to other types of gelatin film which is contributed by higher hydrophobicity constituents due to lower proline and hydroxyproline contents. Apart from that, the melting point and mechanical properties of poultry gelatin films were recorded at higher value compared to mammalian and marine gelatin films due to higher composition of imino acid which has contributed to a stiffer and more rigid gelatin structure. Thus, it can be concluded that poultry gelatin films have better mechanical and light barrier properties in comparison to mammalian and marine gelatin films.

## Figures and Tables

**Table 1:** Physical and mechanical properties of mammalian gelatin-based film.

Gelatin Film			Physical Properties									Mechanical Properties		References
		Thickness (mm)	Color			Light Transmission (nm)		Light Transparency (%)	Water Vapor Transmission Rate (g/m s Pa)	Thermal	Biodegration Rate (%)	Tensile Strength (MPa)	Elongation at Break (EAB) (%)	
			L*	a*	b*	200–280	350–800			T_m_/T_max_ (°C)				
Bovine composite film	Bovine/CMC/ chitosan	-	-	-	-	0.15	-	1.70	1.06–1.84 × 10^−15^	-	4.16–30.49	-	-	[63]
	Bovine /CMC	-	-	-	-	2.50–4.00	0.82–0.65	2.88	1.59 × 10^−4^	-	-	11.80	257	[66]
	Bovine/N-chitin	0.11	-	-	-	0.07–1.11	2.73–4.05	2.50	8.89 × 10^−10^	102.9	-	119.09	-	[38]
	Bovine/0.1,0.2,0.3 corn oil	0.11–0.15	-	-	-	0.06–0.83	1.47–3.02	5.38–5.94	7.68–7.86 × 10^−10^	104.50–113.00	-	28.08–39.47	-	[38]
	Bovine hide/oregano essential oils	0.10–0.13	88.89–89.12	−0.24–0.23	2.02–5.12	-	-	-	0.81–1.21 × 10^−10^	-	-	8.90–14.00	8.30–10.10	[77]
	Bovine hide/lavender essential oils	0.07–0.11	88.02–89.00	−0.60–(−0.29)	4.96–7.23	-	-	-	0.68–1.27 × 10^−10^	-	-	8.80–15.40	4.30–7.60	[77]
	Bovine/*Zataria multiflora* essential oil	-	-	-	-	-	-	-	-	-	-	2.70–4.40	125.00–172.00	[88]
	Bovine/methyl orange indicator	0.05	47.02–80.30	17.11–58.34	−4.17–65.27	-	-	-	6.71–8.28 × 10^−11^	-	-	-	-	[18]
	Bovine/neutral red indicator	0.05	50.63–64.93	16.81–58.01	4.64–25.85	-	-	-	8.58–8.90 × 10^−11^	-	-	-	-	[18]
	Bovine/ bromocresol green indicator	0.05	43.33–91.53	−5.73–(−10.66)	−47.01–31.57	-	-	-	8.62–9.90 × 10^−11^	-	-	-	-	[18]
	Bovine/curcumin extract	0.05–0.06	28.00–89.10	−2.40–35.80	19.10–86.40	-	-	-	0.90–1.20 × 10^−10^	-	-	1.90–3.40	144.30–198.60	[39]
	Bovine/red cabbage extract	0.05–0.06	-	-	-	-	-	-	6.50–12.00 × 10^−11^	-	-	-	-	[39]
	Bovine/CMC/ Chitosan	-	-	-	-	-	-	-	-	-	40.09–85.50	-	-	[89]
	Bovine/carrot residue fiber	-	-	-	-	0.02–0.51	0.03–58.91	-	-	-	-	-	-	[90]
	Bovine/ butterfly pea anthocyanin	0.04	33.00	3.30	−23.00	-	-	-	-	-	-	-	2.10	[71]
Pig gelatin composite film	Pig skin/chitosan	-	90.40	1.10	2.70	-	-	-	-	-	-	-	-	[91]
	Pig skin/eugenol essential oil	-	89.60	3.20	17.00	-	-	-	-	-	-	12.00	59.00	[91]
	Pig skin/ginger essential oil	-	92.40	1.70	4.60	-	-	-	-	-	-	35.00	51.00	[91]
	Pig skin/boldo extract	-	-	-	-	-	-	-	-	-	-	3.20	56.00	[92]
	Pig skin/guarana extract	-	-	-	-	-	-	-	-	-	-	3.80	51.00	[92]
	Pig skin/cinnamon extract	-	-	-	-	-	-	-	-	-	-	3.30	51.00	[92]
	Pig skin/rosemary extract	-	-	-	-	-	-	-	-	-	-	3.30	65.00	[92]
	Porcine skin/furcellaran/pu-erh extract	0.10–0.14	82.98–89.30	−1.18–0.21	14.52–34.40	-	-	-	-	158.40–168.90	-	-	-	[72]
	Porcine skin/furcellaran/green tea extract	0.10–0.12	55.71–79.16	7.80–28.27	28.56–61.09	-	-	-	-	161.80–174.10	-	-	-	[72]
	Porcine skin/furcellaran	-	-	-	-	-	-	-	-	173.50	-	-	-	[72]
Bovine	Bovine hide	-	-	-	-	0–24.41	63.90–88.76	0.59–0.61	3.32–3.56 × 10^−10^	-	-	-	-	[93]
	Bovine hide	0.11	89.07	1.7	4.6	-	-	-	1.46 × 10^−10^	-	-	17.70	-	[77]
	Beef skin (4%, 6% and 8% gelatin concentration)	-	-	-	-	-	-	-	-	-	-	-	-	[25]
	Bovine	0.05	93.35–94.41	−1.07–(−0.79)	2.05–2.70	-	-	-	-	-	-	-	-	[18]
	Bovine	0.05–0.06	93.30–97.30	−1.07–(−0.40)	2.00–5.40	-	-	-	-	-	-	-	-	[39]
	Bovine	-	-	-	-	0.05–2.21	3.61–5.44	2.43	9.68 × 10^−10^	82.80	-	-	4.63	[38]
	Bovine	-	-	-	-	-	-	-	-	60.42	48.81–55.29	-	-	[89]
	Bovine	-	-	-	-	-	-	-	-	-	6.03–30.17	-	-	[63]
	Bovine	-	-	-	-	-	-	-	-	-	-	2.97	-	[94]
	Bovine	0.13	-	-	-	-	-	-	-	-	-	51.68	30.83	[74]
	Bovine	0.04	90.00	−1.27	2.50	-	-	-	8 × 10^−11^	-	-	0.70	0.78	[71]
Pig skin	Pork skin	-	96.33–96.97	−0.27–(−0.39)	2.38–3.22	-	-	-	-	-	-	4.46–10.64	48.01–90.55	[25]
	Pig skin	-	90	1.1	2.2	-	-	-	-	-	-	31	-	[91]
	Pig skin	-	-	-	-	-	-	-	-	-	-	2.4	4.4	[92]
	Porcine	-	-	-	-	-	-	-	-	61.71	-	-	-	[89]
	Porcine	-	-	-	-	-	-	-	-	-	-	3.21	-	[94]
	Porcine	0.11	-	-	-	-	-	-	-	-	-	63.25	-	[74]
	Porcine skin	0.10	-	-	-	-	-	-	-	-	-	-	-	[72]
	Porcine skin	0.70	-	-	-	-	-	-	-	-	-	-	-	[73]
	Pig skin	-	-	-	-	-	-	-	-	66.80	-	-	-	[95]
	Porcine	-	-	-	-	-	-	-	-	87.70	-	-	-	[96]

T_max_: temperature, at which sample lost maximum of its weight; T_m_: melting point. The CIELAB, or CIE L* a* b*, color system represents quantitative relationship of colors on three axes: L* value indicates lightness, and a* and b* are chromaticity coordinates.

**Table 2:** Physical and mechanical properties of marine gelatin-based film.

Gelatin Film			Physical Properties									Mechanical Properties		References
		Thickness (mm)	Color			Light Transmission (nm)		Light Transparency (%)	Water Vapor Transmission Rate (g/m s Pa)	Thermal	Biodegradation Rate (%)	Tensile Strength (MPa)	Elongation at Break (EAB) (%)	
			L*	a*	b*	200–280	350–800			T_m_/T_max_ (°C)				
Marine composite film	Unicorn leatherjacket skin/bergamot essential oil	-	79.59–83.20	0.55–2.10	5.26–6.62	-	-	-	-	-	-	-	-	[97]
	Unicorn leatherjacket skin/lemongrass essential oil	-	83.51–86.72	0.29–0.83	4.16–5.79	-	-	-	-	-	-	-	-	[97]
	Cold fish skin/ silver-copper nanoparticles	-	30.21–52.64	−0.76–2.26	7.74–13.44	-	-	-	-	-	-	-	-	[98]
	Tilapia skin/ epigallocatechin gallate	-	89.06–89.36	−1.39–(−1.35)	1.79–1.88	-	-	-	-	-	-	-	-	[99]
	Tilapia skin/ginger essential oils	-	89.84–90.53	−2.83–(−1.88)	4.00–12.23	0.00–17.85	26.51–86.97	1.60–3.02	1.88–2.61 × 10^−11^	-	-	18.58–35.73	41.70–72.03	[100]
	Tilapia skin/turmeric root essential oils	-	90.04–90.92	−2.98–(−2.20)	6.34–13.43	0.00–0.70	13.44–87.12	1.45–1.63	1.89–2.48 × 10^−11^	-	-	23.34–34.04	42.79–72.08	[100]
	Tilapia skin/plai essential oils	-	90.25–91.11	−3.02–(−1.98)	5.39–11.62	0.00–2.39	14.63–88.23	1.49–2.17	2.45–2.91 × 10^−11^	-	-	17.20–32.06	44.96–74.68	[100]
	Fish skin/ *Ziziphora clinopodioides* essential oil	0.06	87.26	−1.58	13.22	-	-	-	-	-	-	-	-	[80]
	Fish skin/grape seed extract	0.06	55.12	16.77	12.57	-	-	-	-	-	-	-	-	[80]
	Cold water fish skin/ haskap berries extract	0.05	75.55–90.35	1.32–9.09	−4.47–(−1.48)	-	-	-	5.96–7.14 × 10^−11^	-	-	46.70–51.50	2.87–3.69	[50]
	Tilapia skin/bergamot essential oil	-	-	-	-	0.00–19.34	40.34–74.71	4.28–4.45	3.15–3.22 × 10^−11^	-	-	-	-	[101]
	Tilapia skin/kaffir lime essential oil	-	-	-	-	0.00–23.51	47.52–68.32	2.08–2.09	2.95–3.38 x 10^−11^	-	-	-	-	[101]
	Tilapia skin/lemon essential oil	-	-	-	-	0.00–22.26	42.35–66.58	5.31–5.46	2.81–2.85 × 10^−11^	-	-	-	-	[101]
	Tilapia skin/lime essential oil	-	-	-	-	0.00–26.01	49.25–69.37	5.46–5.66	2.91–3.37 × 10^−11^	-	-	-	-	[101]
	Silver carp skin/green tea extract	-	-	-	-	0.01–0.09	6.20–77.80	4.09–4.23	-	-	-	-	-	[102]
	Tuna skin/ Soloyo Grande murta leaves extract	-	-	-	-	-	-	-	7.97 × 10^−13^	-	-	-	-	[103]
	Tuna skin/ Soloyo Grande murta leaves	-	-	-	-	-	-	-	5.08 × 10^−13^	-	-	-	-	[103]
	Cold water fish skin/ *Origanum vulgare* L. essential oil	0.07–0.09	-	-	-	-	-	-	1.35–1.90 × 10^−10^	-	-	3.28–6.72	87.20–151.82	[78]
	Fish skin/ peppermint essential oils	0.12–0.13	-	-	-	-	-	-	-	-	-	-	-	[49]
	Fish skin/ citronella essential oils	0.12–0.13	-	-	-	-	-	-	-	-	-	-	-	[49]
	Tilapia skin/25,50,75 and 100% palm oil	0.06–0.12	-	-	-	-	-	-	-	117.77–123.52	-	-	-	[81]
	Tilapia skin/basil	-	-	-	-	-	-	-	-	74.83–77.92	-	-	-	[104]
	Tilapia skin/citronella	-	-	-	-	-	-	-	-	7.58–83.08	-	-	-	[104]
	Golden carp skin/ palm oil	-	-	-	-	-	-	-	-	107.60	-	-	-	[105]
	Golden carp skin/ squalene rich fraction from shark liver	-	-	-	-	-	-	-	-	110.08–123.79	-	-	-	[105]
	Tilapia skin/basil essential oil	0.06–0.07	-	-	-	-	-	-	-	-	-	14.11–14.66	94.20–127.16	[106]
	Tilapia skin/palm oil	-	-	-	-	-	-	-	-	-	-	11.95–21.81	104.95–143.19	[106]
	Silver carp skin/cinnamon essential oil	-	-	-	-	-	-	-	-	-	-	-	-	[85]
	Carp skin/furcellaran	-	-	-	-	-	-	-	-	-	-	-	68.29	[107]
	Carp skin/furcellaran/rosemary extract	-	-	-	-	-	-	-	-	-	-	-	69.63–75.71	[107]
	Fish bone gelatin/chitosan/tapioca flour	-	-	-	-	-	-	-	-	-	8.48–99.84	-	-	[108]
	Grey triggerfish skin/blood orange peel pectin	0.10	-	-	-	0.01–0.70	12.68–95.70	1.12–1.57	1.54 × 10^−10^	-	-	-	-	[79]
Marine skin	Tilapia skin	-	-	-	-	-	-	-	-	-	-	-	-	[109]
	Tilapia skin	-	90.32	−1.52	1.68	-	-	-	-	-	-	-	-	[99]
	Tilapia skin	-	90.57	−1.58	2.15	0.01–40.73	-	1.20	-	-	-	43.62	-	[100]
	Cold fish skin	-	90.52	−1.30	2.81	-	-	-	-	-	-	-	-	[98]
	Fish skin	0.05	91.42	−2.51	15.88	-	-	-	-	-	-	-	-	[80]
	Cold water fish skin	0.05	94.25	−0.80	−1.68	-	-	-	-	-	-	-	-	[50]
	Silver carp skin	-	-	-	-	-	74.33–89.58	0.05	-	-	-	-	-	[110]
	Silver carp skin	-	-	-	-	0.04–13.80	58.70–85.40	2.86	-	-	-	-	-	[102]
	Fish skin	-	-	-	-	0.01–26.57	-	-	-	-	-	-	-	[93]
	Big-eye snapper skin	-	-	-	-	-	69.30–84.80	-	-	53.14–96.42	-	-	-	[3]
	Brownstripe red snapper skin	-	-	-	-	-	73.20–85.10	-	-	59.89–100.28	-	-	-	[3]
	Tilapia skin	-	-	-	-	-	79.94–88.43	2.14–2.15	-	-	-	-	-	[101]
	Tuna skin	-	-	-	-	-	-	-	6.0 × 10^−13^	-	-	-	-	[103])
	Cold water fish skin	0.06	-	-	-	-	-	-	1.99 × 10^−10^	-	-	10.57	44.71	[78]
	Tilapia skin	0.05	-	-	-	-	-	-	2.54 × 10^−11^	117.43	-	-	44.09	[81]
	Giant catfish skin	-	-	-	-	-	-	-	-	76.50	-	-	-	[111]
	Tilapia skin	-	-	-	-	-	-	-	-	76.92	-	-	-	[104]
	Golden carp skin	-	-	-	-	-	-	-	-	124.45	-	-	-	[105]
	Warm-water Tilapia skin	-	-	-	-	-	-	-	-	-	-	-	-	[25]
	Cold water fish skin	-	-	-	-	-	-	-	-	-	-	-	76.73	[112]
	Fish skin	0.12	-	-	-	-	-	-	-	-	-	-	-	[49]
	Silver carp skin	-	-	-	-	-	-	-	-	-	-	29.03	-	[85]
	Cold water fish skin	-	-	-	-	-	-	-	-	-	-	-	2.96	[50]
	Grey triggerfish skin	0.08	-	-	-	0.01	28.36–90.50	1.09	2.05 × 10^−10^	-	-	6.23	10.47	[79]

T_max_: temperature, at which sample lost maximum of its weight; T_m_: melting point. The CIELAB, or CIE L* a* b*, color system represents quantitative relationship of colors on three axes: L* value indicates lightness, and a* and b* are chromaticity coordinates.

**Table 3:** Physical and mechanical properties of poultry gelatin-based film.

Gelatin			Physical Properties									Mechanical Properties		References
		Thickness (mm)	Color			Light Transmission (nm)		Light Transparency (%)	Water Vapor Transmission Rate (g/m s Pa)	Thermal	Biodegradation Rate (%)	Tensile Strength (MPa)	Elongation at Break (EAB) (%)	
			L*	a*	b*	200–280	350–800			T_m_/T_max_ (°C)				
Poultry composite film	Chicken feet gelatin/25% glycerol	0.06	90.77	−1.30	3.01	-	72.48–87.58	1.08	2.04 × 10^−11^	-	-	44.86	15.99	[86]
	Chicken feet gelatin/35% glycerol	0.06	91.29	−1.40	3.18	-	66.75–85.62	1.10	2.14 × 10^−11^	-	-	34.20	33.30	[86]
	Chicken feet gelatin/sugarcane bagasse	0.07–0.09	-	-	-	-	-	-	-	-	-	-	-	[86]
	Chicken skin gelatin /CMC	-	-	-	-	2.07–4.00	0.59–1.08	2.92	-	126.93	-	5.53	310	[66]
	Chicken skin/rice flour	-	-	-	-	0.06–3.89	15.29–50.96	2.45–3.06	6.83 × 10^−10^–1.39 × 10^−9^	1st peak: 49.81–51.48 2nd peak: 124.97–129.25	-	2.08–2.91	58.45–79.31	[8]
	Chicken skin/5–20% glycerol	-	-	-	-	0.02–4.29	40.50–78.90	0.81–3.97	4.86–6.67 × 10^−12^	-	-	1.75–3.64	106.43–148.33	[64]
	Chicken skin/CMC/*Centella asiatica*	-	-	-	-	0.00–0.02	0.03–9.24	0.71–0.86	1.11–1.13 × 10^−4^	130.11–131.31	-	4.50–5.00 × 10^−2^	271.17–281.00	[16]
	Chicken skin/CMC	-	-	-	-	0.00–0.42	4.07–7.43	0.82	1.03 × 10^−4^	124.38	-	3.00 × 10^−2^	223.05	[16]
Poultry skin	Chicken skin	-	-	-	-	2.59–2.93	3.98–11.98	-	-	134.22	-	0.98	561	[66]
	Chicken skin	-	-	-	-	0.16–5.89	41.86–51.97	1.94	5.94 × 10^−10^	49.51	-	1.54	48.33	[8]
	Chicken skin	-	-	-	-	0.03–4.48	61.77–80.63	0.77	4.17 × 10^−12^	-	-	33.66	3.87	[64]
	Chicken skin	-	-	-	-	-	-	-	-	76.26	-	-	-	[94]

T_max_: temperature, at which sample lost maximum of its weight; T_m_: melting point. The CIELAB, or CIE L* a* b*, color system represents quantitative relationship of colors on three axes: L* value indicates lightness, and a* and b* are chromaticity coordinates.
